# The complete mitochondrial genome of threadfin butterflyfish, *Chaetodon auriga* (Chaetodontiformes: Chaetodontidae) and phylogenetic analysis

**DOI:** 10.1080/23802359.2022.2136982

**Published:** 2022-11-04

**Authors:** Maheshkumar Prakash Patil, Jong-Oh Kim, Yu-Jin Lee, Yong Bae Seo, Jin-Koo Kim, Gun-Do Kim

**Affiliations:** a Industry-University Cooperation Foundation, Pukyong National University, Busan, Republic of Korea; b Department of Microbiology, Pukyong National University, Busan, Republic of Korea; c School of Marine and Fisheries Life Science, Pukyong National University, Busan, Republic of Korea; d Department of Marine Biology, Pukyong National University, Busan, Republic of Korea; e Research Institute for Basic Science, Pukyong National University, Busan, Republic of Korea

**Keywords:** *Chaetodon auriga*, threadfin butterflyfish, Chaetodontidae, mitogenome, phylogenetic analysis

## Abstract

*Chaetodon auriga* (Forsskal, 1775) belongs to the family Chaetodontidae and the order Chaetodontiformes. Here, we report the complete mitochondrial genome of *C. auriga* assembled using the Illumina MiSeq platform. The circular mitochondrial genome of *C. auriga* is 16,527 bp long, has an A + T content of 54.53%, and contains 37 genes (13 protein-coding genes, 22 tRNA genes, and 2 rRNA genes), and a non-coding region. The overall nucleotide composition was A: 28.19%, T: 26.34%, G: 16.27%, and C: 29.20%. The mitochondrial genome of *C. auriga* contributes to revealing the phylogenetic relationships among species of the Chaetodontidae family.

*Chaetodon auriga* also known as threadfin butterflyfish belongs to the family Chaetodontidae and the order Chaetodontiformes, found in tropical coral reefs and widely distributed throughout the Indo-Pacific region. *Chaetodon* is a widespread genus, and several studies have proposed closely related species based on morphological appearance and selected mitochondrial genes (Hsu et al. 2007). However, there is no report on the complete mitochondrial genome of *C. auriga*, as a result, in this study, we focused on the characterization of complete mitochondrial genome features and their phylogenetic relationship with other related species. The complete mitochondrial genome of *C. auriga* will serve as a valuable tool for understanding the phylogenetic relationship and evolutionary history of Chaetodontidae.

The *C. auriga* specimen (Figure 1) was captured from the coast of Kamikatetsu in Japan (28°27′34.99′′N 129°94′58.56′′E) and deposited at the Marine Fish Resources Bank of Korea (MFRBK) in Pukyong National University (PKNU), Busan, Republic of Korea (Dr. Jin-Koo Kim, taengko@pknu.ac.kr) under the voucher number KAUM-148105. Total genomic DNA was extracted from muscle tissues using the DNeasy Blood and Tissue Kit (Qiagen, Germany) according to the manufacturer’s instructions. The DNA library was generated using the TrueSeq Nano DNA Kit and sequenced on the Illumina platform with 150 bp paired-end reads (Illumina, HiSeq 2500, San Diego, CA, USA). Next, the SPAdes v3.13.0 assembly tool (Bankevich et al. 2012) was used for *De novo* assembly and the MitoFish web server (http://mitofish.aori.u-tokyo.ac.jp/) was used for complete mitochondrial genome sequence annotation (Iwasaki et al. 2013).

**Figure 1.**
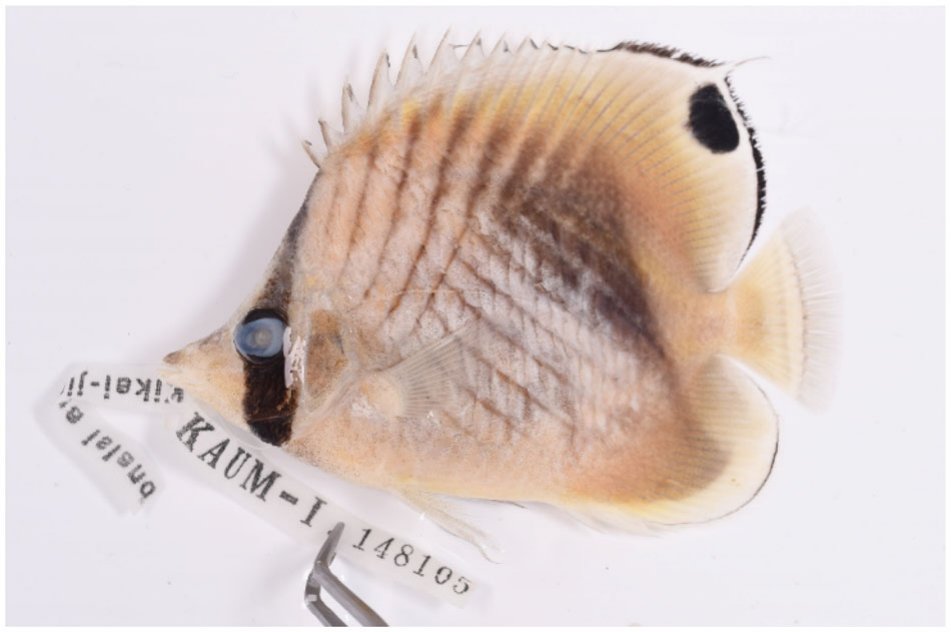
Specimen image of *Chaetodon auriga* (threadfin butterflyfish) having characteristic black vertical band runs through the eye and dorsal fin has a black spot with a trailing filament.

The assembled mitochondrial genome of *C. auriga* (16,527 bp in length) is available in the NCBI GenBank database under the accession number ON843633. The complete mitochondrial genome of *C. auriga* is a closed circular, with an overall nucleotide composition of A: 28.19%, T: 26.34%, G: 16.27%, and C: 29.20%, with a slight A + T bias (54.53%), and contains 13 protein-coding genes (*ATP6*, *ATP8*, *Cytb*, *COX1*, *COX2*, *COX3*, *ND1*, *ND2*, *ND3*, *ND4*, *ND4L*, *ND5*, *ND6*), 2 ribosomal RNA genes (*12S rRNA*, *16S rRNA*), 22 transfer RNA and a non-coding region (Figure 2). Among the 38 sequence elements, the *ND6* gene and eight tRNA (*Ile*, *Trp*, *Ala*, *Asn*, *Cys*, *Try*, *Ser*, *Glu*, and *Pro*) genes were located on the light strand, whereas others were located on the heavy strand. The *12S*, and *16S rRNA* genes of *C. auriga* are positioned between the *tRNA-Phe* and *tRNA-Leu* genes, separated by the *tRNA-Val* gene, similar to the typical mitochondrial genome of vertebrates. The features mentioned before were in accordance with the typical Chaetodontidae fish mitogenome.

**Figure 2.**
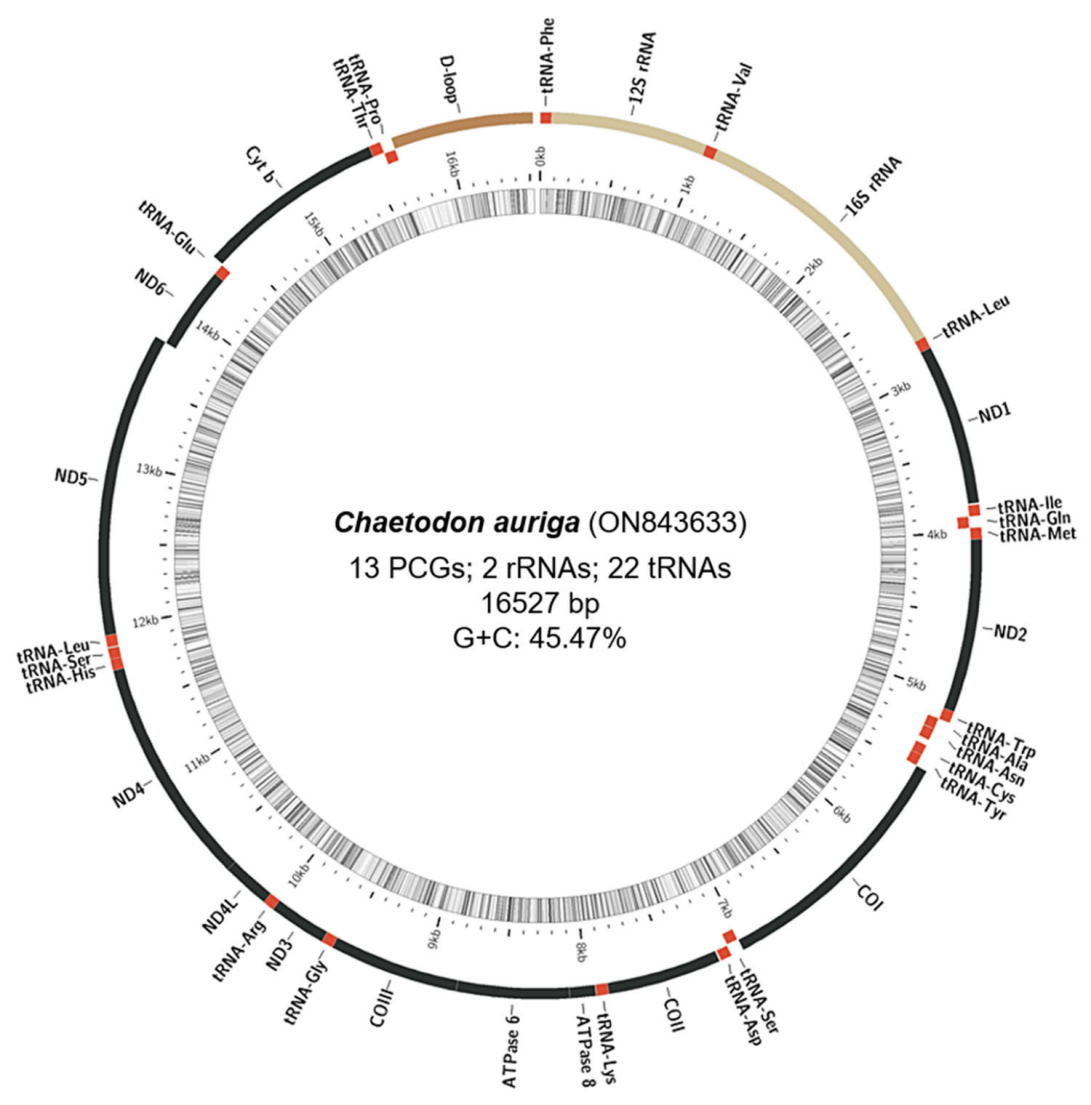
The circular-mapping mitochondrial genome of *C. auriga* prepared using the MitoFish web-server. Genes outside the circle are transcribed clockwise, whereas those inside are transcribed counterclockwise. PCGs: protein-coding genes.

The phylogenetic relationships of *C. auriga* (ON843633) and 10 other Chaetodontidae species were studied using the complete mitochondrial genome sequences. One species, *Salvelinus malma* (MF680544) of the Salmonidae was chosen as the outgroup, according to Yang et al. (2021). Mitochondrial genome sequence alignment and phylogenetic analysis were conducted using MEGA11 v11.0.8 based on the Maximum-likelihood approach (Tamura–Nei model) with 1000 bootstraps replications (Tamura et al. 2021). All 11 species from Chaetodontidae form one clade with full support on all the nodes. The eight Chaetodon species formed a monophyletic clade sister to the clade consisting of three *Heniochus* species and one *Forcipiger* species (Figure 3). The topology of the phylogenetic tree generated in this study suggests that *C. auriga* is positioned in the sister branch to *Chaetodon weibeli* and *Chaetodon auripes* with a supporting bootstrap value of 100 and has a very close relationship to other species in the family Chaetodontidae. This study describes the complete mitochondrial genome of *C. auriga* as well as their phylogenetic relationship within the Chaetodontidae family. To better understand the phylogenetic relationship among Chaetodontiformes species, the mitochondrial genome study within the order must be expanded.

**Figure 3.**
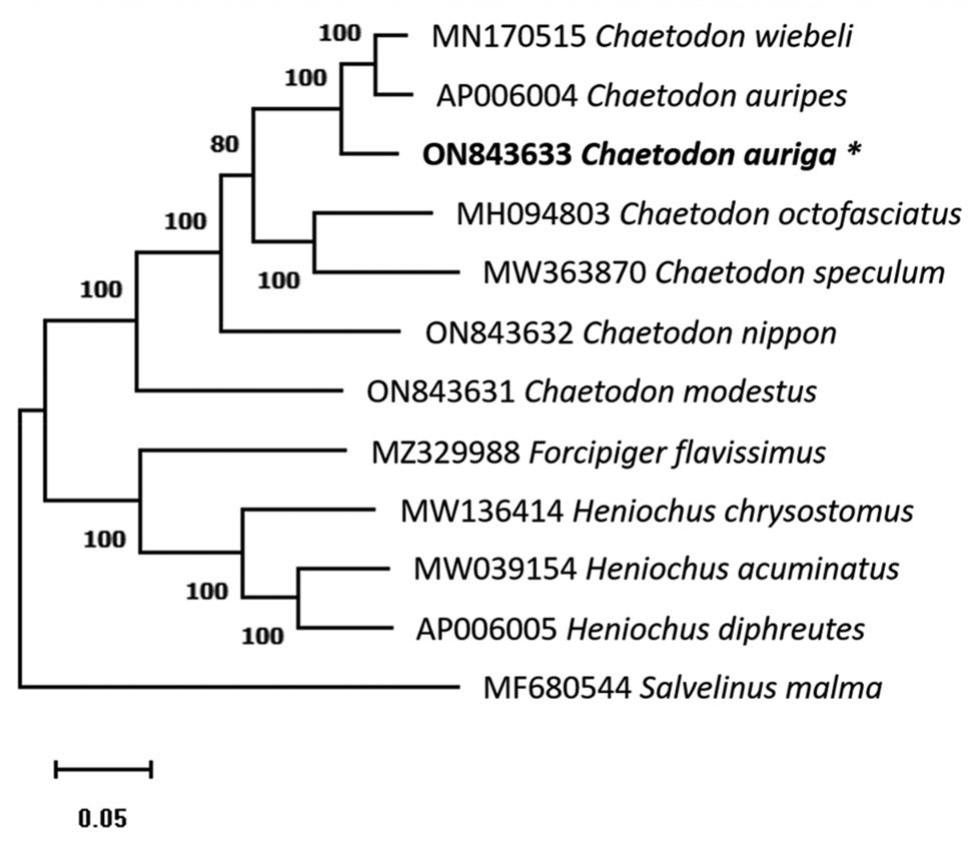
Maximum-likelihood phylogenetic tree reconstruction of *Chaetodon auriga* in Chaetodontidae based on whole mitochondrial genome data. The GenBank accession numbers of all mitochondrial genomes utilized for phylogenetic analysis are followed by species names, and the newly described species in this work are marked with an asterisk next to their names. The number above the branches denotes maximum probability bootstrap values.

## Data Availability

The genome sequence data that support the findings of this study are openly available in GenBank of NCBI at (https://www.ncbi.nlm.nih.gov/nuccore/ON843633) under accession no. ON843633. The associated BioProject, BioSample, and SRA numbers are PRJNA854786, SAMN29447702, and SRR19913669, respectively.
